# Do We Have a Knowledge Gap with Our Patients?—On the Problems of Knowledge Transfer and the Implications at the End of Life

**DOI:** 10.3390/ijerph22020247

**Published:** 2025-02-10

**Authors:** Nils Heuser, Hendrik Heers, Martin Gschnell, Fabian Urhahn, Severin Schrade, Christian Volberg

**Affiliations:** 1 Department of Anaesthesiology and Intensive Care Medicine, Philipps University of Marburg, 35043 Marburg, Germany; 2 Department of Urology and Paediatric Urology, University Hospital RWTH Aachen, 52074 Aachen, Germany; 3 Department of Urology, Philipps University of Marburg, 35043 Marburg, Germany; 4 Department of Dermatology und Allergology, Philipps University of Marburg, 35043 Marburg, Germany; 5 Department of Urology, Landesklinikum Mistelbach-Gänserndorf, 2130 Mistelbach, Austria; 6 Department of Cardiology, Kreisklinik Ebersberg, 85560 Ebersberg, Germany; 7 Research Group Medical Ethics, Faculty of Medicine, Philipps University of Marburg, 35043 Marburg, Germany

**Keywords:** end-of-life care, oncology, communication, cancer care, palliative care

## Abstract

Background: Cancer patients are often unaware of their exact diagnosis, stage of disease, and prognosis. This affects their treatment, quality of life, and end-of-life decisions. In this study, patients with skin and urological cancers were asked about their level of knowledge about their disease and its treatment in order to highlight this problem and describe possible effects on end-of-life decisions. Methods: 150 patients with advanced skin cancer and 88 patients with advanced urological cancer were interviewed using a structured questionnaire at a German university hospital. Descriptive and statistical analysis of the data were performed. The significance level was set at alpha ≤ 0.05. Results: 67% of skin cancer patients could not state their exact stage. Of these, younger patients (<60 y) were more likely to state their stage correctly (*p* = 0.017). All of those patients knew about their therapy. A total of 56 patients had distant and 143 had local metastasis. The majority was aware of that (84%, 78%). Also, 95% of the urological cancer patients stated that they knew their stage of disease, but not a single patient could tell it correctly. All urological patients knew about the presence of metastasis. A total of 30% of urological cancer patients were unaware of their tumor therapy, and one patient stated that he did not receive any treatment, even though he did. The majority of patients could not correctly name their exact therapy. Conclusions: In the patients observed, it was found that many of them were unaware of their stage of disease, which can have a huge impact on their end-of-life decisions, such as the type of treatment they want. Many patients were also unaware of their own treatment. There is a risk that false hopes of cure may be attached to therapies and that, as a result, patients may be less likely to opt for palliative care with a focus on maintaining quality of life.

## 1. Background

Cancer is one of the main reasons for the use of palliative care at the end of life in Germany, accounting for approximately 86% [1]. This is reflected in the fact that the current guidelines for palliative care in Germany explicitly refer to patients with incurable cancer [2].

However, numerous studies from different countries around the world show that cancer patients are often unaware of their exact diagnosis, status, or prognosis of their disease [3,4,5,6,7,8,9,10,11,12]. This has been described not only in cancer per se but also in other chronic diseases and in relation to specific tumor diseases such as breast cancer or sarcoma [4,5,13].

This is a serious problem because patients can benefit in many ways from knowing their exact diagnosis and prognosis. This can be demonstrated, for example, in relation to their health-related quality of life [14]. In addition, informed patients have more realistic expectations about their life expectancy and are more likely to choose not to undergo life-prolonging therapies and interventions [15,16]. No negative effects of comprehensive information about diagnosis or prognosis on patients’ emotional well-being or on the doctor-patient relationship have been observed [15]. 

It is not clear whether this knowledge gap between clinicians and patients is solely due to a lack of communication. It seems to be a multifactorial problem. For example, there also seems to be an association between low levels of education and lack of awareness of the diagnosis and prognosis of the disease, and there are cultural differences in coping with serious illness [3,5,11,12]. It is well known and understandable that communicating an unfavorable prognosis to a patient with advanced cancer is one of the most difficult conversations to have as a clinician [17]. However, these discussions are of great importance in preventing patients from having unrealistic expectations about their prognosis and treatment, thus enabling them to accept the disease and receive treatment with a focus on maintaining their quality of life. This is particularly important as cancer patients are surviving longer as a result of new, modern treatments, which may also lead to longer palliative care [18]. Knowledge of the diagnosis and prognosis also affects treatment. It has been shown that patients who did not know their exact diagnosis often did not know why they were taking their medication or undergoing chemotherapy, with the result that they placed false, curative hopes in this therapy [3,19].

In this study, we specifically asked dermatological and urological cancer patients with different tumor entities about their diagnosis, stage of disease, and treatment to investigate whether this problem is also reflected in this patient population and what conclusions can be drawn about the treatment of these patients in the last stage of life.

## 2. Methods

The present studies were submitted to the Ethics Committee of the School of Medicine at Philipps University of Marburg for review. They were approved under reference numbers 159/20 (dermato-oncology study, 20 October 2020) and 34/21 (uro-oncology study, 8 March 2021) and registered in the German Clinical Trials Register (dermato-oncology: DRKS00023492 (3 November 2020), uro-oncology: DRKS00025957 (20 August 2021)). Data from both studies have already been published formerly [20,21]. These primary studies addressed the preferences of dermatology and urology patients with advanced cancer at the end of life and the extent to which arrangements regarding those preferences were made and communicated. For the present evaluation, an additional subgroup analysis was performed with regard to the research question. As these were originally two different studies with similar but not identical questionnaires, the collected data were not always comparable in detail.

Eligible skin cancer patients were adult patients of the Marburg Skin Tumor Centre with advanced skin cancer (according to TNM AJCC 2017: malignant melanoma clinical stage III + IV, Merkel cell carcinoma clinical stage III + IV, squamous cell carcinoma clinical stage III + IV) who were seen for routine follow-up between 2 November 2020 and 2 September 2021. All patients routinely received their medical report with their exact diagnosis after their follow-up.

The uro-oncology study was conducted with patients treated at the Oncology Clinic of the Department of Urology at Marburg University Hospital from March to December 2021. The study included patients undergoing medical tumor therapy for metastatic or inoperable prostate cancer, urothelial carcinoma, or renal cell carcinoma. They were asked to participate in the survey while waiting for their consultation.

Both questionnaires (for original questionnaires see Supplemental Materials) covered initial diagnosis, tumor stage, metastases, and treatment. A research assistant was available for participants to answer questions or help with the questionnaire. For some questions, responses were compared with the patient file for validation. Consent has been obtained from all study participants.

Data analysis and management were performed using Microsoft^®^ Excel version 16.68, R-Studio, and SPSS^®^ Statistics version 27 (IBM Corp., Armonk, NY, USA). Several qualitative characteristics were checked for dependency using Chi^2^-tests. The significance level was set at alpha ≤ 0.05.

## 3. Results

For the dermatology study, a total of 166 patients were contacted over a period of 10 months to participate in the survey, of whom 150 (90%) agreed. The mean age of the sample population was 65 years (standard deviation [SD] = 14.4 [range 22–97]), and the majority of patients were male (57%, *n* = 85). The vast majority (88%, *n* = 132) had malignant melanoma, followed by squamous cell carcinoma (8%, *n* = 12) and Merkel cell carcinoma (4%, *n* = 6). A total of 62% of patients had AJCC/UICC stage III (*n* = 93) and 38% had stage IV (*n* = 57) cancer. Additional demographic data are shown in Table 1. The stage of their disease was correctly recalled by 33% of patients (*n* = 49); the remaining 67% (*n* = 101) could not correctly state the stage of their disease (see Table 2). A total of 56 patients had distant metastasis, and 143 had local or lymph node metastasis. The vast majority was aware of that circumstance (84% of distant, 78% of local/lymph node; see Table 2). Patients younger than 60 years of age were significantly more likely to correctly name the stage of their disease (*p* = 0.017; see Table 3). There was no significant correlation between entity and knowledge of stage (*p* = 0.17) nor for the factors of gender, education, and the type of nursing care (see Table 3). Moreover, 46.7% (*n* = 70) of the patients received therapy. All patients were aware of their therapy.

A total of 88 patients participated in the urological survey. Most patients were male (86%, *n* = 76). Full demographic details are shown in Table 4. A total of 47% of patients (*n* = 41) had prostate cancer, followed by renal cell carcinoma (40%, *n* = 35) and urothelial carcinoma (14%, *n* = 12). Most patients stated that they knew their exact tumor stage (95%, *n* = 84), but no patient could actually name it correctly (see Table 5). Therefore, we were not able to analyze factors influencing the patients’ knowledge of their tumor stage. A total of 87 patients had metastatic disease. Each of these patients was aware of this. The exact entity and knowledge of stage and metastasis are shown in Table 5. About 29.5% of patients (*n* = 26) were unable to state the type of therapy they were receiving. One patient reported not receiving any therapy, although he was treated. Fewer patients reported receiving immunotherapy, chemotherapy, or hormone therapy than actually did. There was no significant correlation between tumor entity and knowledge of therapy (*p* = 0.69, see Table 6). The exact details of the patients’ treatment are shown in Table 6.

## 4. Discussion

Medical interventions and therapies always require both an indication and the patient’s consent [22]. To give informed consent, the patient must be fully informed and aware of all risks, side effects, and alternative treatment options [23]. We were able to show that cancer patients are often not sufficiently aware of their disease and its consequences. The question is whether a patient who is not informed about the stage of their disease and its prognosis can be considered an informed patient at all. Manson and O’Neill, in their work “Rethinking informed consent in bioethics“, state that uninformed consent cannot be considered consent, even if the patient fully agrees to the proposed therapy:

“If a patient or research subject remains uninformed or under-informed about what others (researchers, clinicians) propose or request, then however eagerly or fully he appears to agree to their proposals, those indications of agreement do not count as giving consent and do not license others to act as they propose.” [24].

They specify this idea by emphasizing that the consent of patients who cannot adequately understand the proposals made to them is flawed and does not legitimize the proposed action [24]. But is not knowledge of one’s own disease, its stage, and its prognosis necessary to adequately understand one’s own situation and thus to be able to decide which treatment alternative is preferred? In our patient cohort, we found that more than two-thirds of all skin cancer patients surveyed did not know the stage of their disease (see Table 2). None of the urological patients were able to correctly state the stage of their disease (see Table 5). All urological patients and most of the dermatological patients were aware of metastasis (see Table 2 and Table 5). It can be assumed that metastasis is easier to understand than the complex classification of tumor stages. But can we really speak of informed patients and a legitimization of therapy in these cases?

Lack of knowledge about the disease and its stage also has an impact on the therapy used. While all the skin cancer patients knew about their treatment, this did not apply to the urology patients. Given that the stage of the tumor has a significant influence on the type of treatment, it is not surprising that most urological patients who did not know their stage were also unable to name their specific treatment (see Table 6). A total of 30% of them were unaware of their treatment, and one patient stated that he was not receiving treatment even though he was. Not knowing what treatment they are receiving can lead to false hope, as has been shown for chemotherapy in patients with advanced cancer. The patients were not aware of the palliative intent of this therapy [19]. In addition, patients with a low health literacy receiving chemotherapy are more likely to have severe drug reactions [25]. This may further limit the ability of these patients to make an informed decision for or against treatment. The influence of knowledge about the stage of disease and about their treatment is also demonstrated by the fact that patients with advanced cancer who knew their stage were more likely to opt out of life-prolonging therapies [15,16]. There are also studies showing that such informed patients have a higher health-related quality of life and a better understanding of the terminal nature of their prognosis [6,14]. According to the principles of palliative care, it is this quality of life that should be the focus of attention for patients with incurable cancer and should guide medical treatment [26].

But why is there such an information gap between patients and their doctors? It is likely that the problem is multifactorial. First, end-of-life discussions are a major challenge for physicians and are among the most difficult discussions to have in medical practice [17]. Doctors, carers, and relatives face many barriers to such discussions, which is why they are often avoided [27]. Not only is it challenging to break down the medical facts into a language that can be understood by patients and relatives—an increasing number of whom have a different primary language than that of the medical professionalist—but those conversations also require enough time and a setting without interruptions and disturbances, which is hard to achieve in the real-world clinical situation. On the other hand, doctors are often afraid of losing patients’ trust or control in their communication with patients by giving bad news, despite other studies showing that patients expect their doctors to be honest and wish to be adequately informed by them [28,29]. However, there are also patient-related factors that can lead to such a discrepancy. For example, studies show that patients with terminal cancer are less likely to want to be informed of their exact diagnosis than patients with earlier-stage cancer [12]. Other investigations demonstrated a link between older age and a lack of understanding of one’s disease and that the preferences of adults concerning end-of-life issues differ by age [3,12,30]. We were able to show similar data for skin cancer patients, as younger patients were more likely to correctly state the stage of their disease (see Table 3). This was not the case for the urology patients, as none of them were able to give the exact stage of their tumor. There also seems to be a correlation between a low level of education and knowledge about the disease, but we have not been able to show this with our data comparing patients with and without a university degree (see Table 3) [5,12]. But not only individual factors could have an influence on this topic. Also, differences in culture and ethnicity affect the handling of end-of-life decisions and the dealing with serious illness [11,31,32,33].

In summary, it is likely that barriers to informing patients about (poor) diagnoses, prognoses, and treatment options can be avoided by listening to patients’ preferences and communicating adequately. With regard to the difficulties associated with talking about dying and death, it may be useful to use tools such as the DEOLD-FI(r) questionnaire to systematically record problems in communicating with patients and relatives [34,35]. Only by highlighting these barriers can we overcome them and improve communication with people at the end of life. This is essential if patients are to be able to make informed decisions about end-of-life treatment.

The variety of possible causes that can lead to a knowledge gap between the healthcare team and the patient is a powerful illustration of the complexity of this problem and the complexity of treating patients with a terminal illness in general. In summary, however, every patient has the right to know the exact status and prognosis of their condition to make informed decisions about the management of their debilitating condition. Most patients want to be informed about their disease and treatment, and no patient should be denied this, as it has been shown that patients benefit from knowledge of their disease by having realistic expectations, being able to make informed decisions, and having a better quality of life [14,15,16].

## 5. Conclusions 

Our data show that patients with oncological diseases are often unaware of the stage of their disease and the treatment procedures used. This can lead to problems in treatment and advance care planning. It is particularly important for palliative care patients to discuss key aspects of the end of life. Therefore, clinicians should discuss with patients at regular intervals during treatment what therapies are being used and what the prognosis of the disease is. Aspects of advance care planning should be integrated into treatment.

## Figures and Tables

**Table 1:** Skin Cancer Patients. Demographic Data.

	*n*	%
**Total**	150	100
**Gender**		
Male	85	57.7
Female	65	43.3
**Age (a)**		
<40	7	4.7
40–59	38	25.3
60–79	80	53.3
>79	25	16.7
**Nursing Care**		
No Care	121	80.7
Out-Patient Nursing Service	18	12
Relatives	9	6
Nursing Home	1	0.7
Private Nurse	1	0.7
**Therapy**		
Immunotherapy	52	34.7
Chemotherapy	2	1.3
Radiotherapy	1	0.7
Targeted Therapy	11	7.3
No Therapy	80	53.3
Immunotherapy and Radiotherapy	4	2.7

**Table 2:** Skin Cancer Patients. Entity and Awareness of Stadium/Metastasis.

	*n*	%
**Total**	150	100
**Entity**		
Malignant Melanoma	132	88
Squamous Cell Carcinoma	12	8
Merkel Cell Carcinoma	6	4
**Stadium (AJCC/UICC)**		
III	93	62
IV	57	38
**Awareness of Stadium (total)**		
Yes	49	32.7
No	101	67.3
	**Distant Metastasis (*n*)**	**Local Metastasis/Lymph Node Metastasis (*n*)**
**Total (*n*)**	56	143
**Awareness of Metastasis**		
Yes	47 (83.9%)	111 (77.6%)
No	9 (16.1%)	32 (22.4%)

**Table 3:** Skin Cancer Patients. Awareness of Stadium in relation to different factors.

Awareness of Stadium	<60 (y)	≥60 (y)	*p*
Yes (*n*)	21	28	0.017
No (*n*)	24	77	
	**Entity**		
***Malignant Melanoma***	***n***	**%**	0.17
Yes	46	34.8	
No	86	65.2	
***Squamous Cell Carcinoma***			
Yes	1	8.3	
No	11	91.7	
***Merkel Cell Carcinoma***			
Yes	2	33.3	
No	4	66.7	
	**Gender**		0.14
	**Male**	**Female**	
Yes (*n*)	25	24	
No (*n*)	60	41	
	**Education**		0.07
	University Degree	No University Degree	
Yes (*n*)	17	21	
No (*n*)	32	80	
	**Nursing Care**		
	Awareness: Yes (*n*)	Awareness: No (*n*)	0.19
No Care	45	76	
Out-Patient Nursing Service	3	15	
Relatives	1	8	
Nursing Home	0	1	
Private Nurse	0	1	

**Table 4:** Urologic Oncology Patients. Demographic Data.

	*n*	%
**Total**	88	100
**Gender**		
Male	76	86
Female	12	14
**Age**		
<70	42	48
>70	46	52
**Nursing Care**		
No	55	62
Yes	33	38

**Table 5:** Urologic Oncology Patients. Entity and Awareness of Stadium/Metastasis.

Entity		
Prostate Cancer	41	46.6
Renal Cell Carcinoma	35	39.8
Urothelial Carcinoma	12	13.6
**Awareness of Stadium (Patients’ Response)**		
Yes	84	95.5
No	4	4.5
**Awareness of Stadium (actually)**		
Yes	0	0
No	88	100
**Metastasis (Patients‘ Response)**		
Yes	87	100
No	0	0
**Metastasis (actually)**		
Yes	87	98.9
No	1	1.1

**Table 6:** Urologic Oncology Patients. Awareness of Therapy in general and according to Tumor Entity.

	Patients’ Response		Actual Therapy	
	*n*	%	*n*	%
**Unaware of Therapy**	26	29.5	-	-
**No Therapy**	8	9.1	7	8
**Immunotherapy**	27	30.7	36	40.9
**Chemotherapy**	10	11.4	16	18.2
**Targeted Therapy**	2	2.3	1	1.1
**Hormone Therapy**	15	17	28	31.8
**Tumor Entity**	**Wrong**	**Right**	**Unaware**	**Total**
**Prostate Carcinoma (*n*)**	5	23	13	41
Proportion of Patients with Prostate Carcinoma (%)	12.2	56.1	31.7	100
Proportion of total Wrong/Right/Unaware Answers (%)	71.4	41.8	50	46.6
**Renal Cell Carcinoma (*n*)**	2	24	9	35
Proportion of Patients with Renal Cell Carcinoma (%)	5.7	68.6	25.7	100
Proportion of total Wrong/Right/Unaware Answers (%)	28.6	43.6	34.6	39.8
**Urothelial Carcinoma (*n*)**	0	8	4	12
Proportion of Patients with Urothelial Carcinoma (%)	0	66.7	33.3	100
Proportion of total Wrong/Right/Unaware Answers (%)	0	14.5	15.4	13.6
**Total (*n*)**	7	55	26	88
Proportion of all Patients (%)	8	62.5	29.5	100
Proportion of total Wrong/Right/Unaware Answers (%)	100	100	100	100

## Data Availability

The original contributions presented in this study are included in the article/Supplementary Materials. Further inquiries can be directed to the corresponding author.

## Supplementary Materials

The following supporting information can be downloaded at: https://www.mdpi.com/article/10.3390/ijerph22020247/s1, Title: Original questionnaires (in German).
