# Supplementary material for: Do We Have a Knowledge Gap with Our Patients?—On the Problems of Knowledge Transfer and the Implications at the End of Life
Source: Int J Environ Res Public Health. 2025 Feb 10;22(2):247. doi: 10.3390/ijerph22020247 (PMC11855585; doi:10.3390/ijerph22020247)
Supplement: Supplementary file 1 [file ijerph-22-00247-s001.zip › ijerph-3433600-supplementary.pdf]

Probanden ID:

Philipps Universität Marburg		UKGM Universitätsklinikum Giessen und Marburg
	Studienleitung	
**Dr. Hendrik Heers**<br>Oberarzt<br>Klinik für Urologie und Kinderurologie, UKGM Marburg<br>Baldingerstraße, 35032 Marburg<br>☎ 06421-58 - 69405	**Dr. Christian Volberg**<br>Klinik für Anästhesie und Intensivtherapie, AG Klinische Ethik<br>Mitglied des klin. Ethikkomitees<br>Baldingerstraße, 35032 Marburg<br>☎ 06421-58-64934	**Prof. Dr. Astrid Morin**<br>Oberärztin<br>Klinik für Anästhesie und Intensivtherapie, Palliativmedizin<br>Baldingerstraße, 35032 Marburg<br>☎ 06421-58-65971

Datum des Interviews:

Fragebogen

Bitte beantworten Sie folgende Fragen, indem Sie ein Kreuzchen bei der zutreffendsten Antwort setzen.

1. Was ist Ihr Geschlecht?

- o weiblich
- o männlich
- o divers

2. Wie alt sind Sie?

3. Wie alt waren Sie, als bei Ihnen die Tumordiagnose gestellt wurde?

4. An welchem urologischen Tumor leiden Sie?

o Prostatakarzinom
o Nierenzellkarzinom
o Urothelkarzinom (z.B. Harnblasenkarzinom)

5. Kennen Sie Ihr Tumorstadium?

- o Ja, und zwar:__________________
- o Nein

6. Haben oder hatten Sie Metastasen (Absiedelungen des Tumors)?

- o Ja
- o Nein
- o Ich weiß es nicht

7. Haben oder hatten Sie noch eine weitere Tumorerkrankung?

- o Ja, und zwar:___________________
- o Nein

8. Leiden Sie unter den aufgeführten Symptomen?

o Nein
o Ja und zwar (mehrere Antworten möglich):

- o Schmerzen
- o Luftnot
- o Harnwegsinfekte
- o Makrohämaturie (Blut im Urin)
- o sexueller Unlust/Problemen
- o Übelkeit
- o Durchfälle/Verstopfung
- o Schlafstörungen
- o ausgeprägte Tagesmüdigkeit (Fatigue)
- o offene Wunden
- o Angst
- o sonstige: ________________

9. Belasten Sie diese Symptome?

o Ja
o Nein
o Ich weiß es nicht

10. Erhalten Sie aktuell eine tumorspezifische Therapie (z.B. Immun-, Chemo-, Strahlen- oder zielgerichtete Therapie)?

- o Ja, und zwar:____________________
- o Nein
- o Ich weiß es nicht

11. Meine Nationalität ist

- o Deutsch
- o Sonstige:_______________________

12. Wie ist Ihr Familienstand?

- o Ledig
- o In fester Beziehung
- o Verheiratet/ in eingetragener Lebenspartnerschaft
- o Verwitwet
- o Geschieden/ aufgehobene Lebenspartnerschaft

13. Haben Sie Kinder?

o Ja, Anzahl:
o Nein

14. Wo leben Sie?

- o Im eigenen Haushalt
- o Bei Kindern bzw. Verwandten
- o Im betreuten Wohnen
- o Im Pflegeheim/Seniorenresidenz (weiter bei 15.)
- o Sonstiges: ________________________

15. Wie viele Personen leben ständig in Ihrem Haushalt (Sie selbst eingeschlossen)?

16. Erhalten Sie pflegerische Unterstützung?

- o Nein
- o Ja, durch Angehörige
- o Ja, durch einen ambulanten Pflegedienst
- o Ja, durch den Pflegedienst im Pflegeheim
- o Ja, durch eine persönliche Pflegekraft
- o Ja, durch:___________________________

17. Welche Wohnlage trifft auf Sie zu?

- o Großstadt (über 100.000 Einwohner)
- o Mittelstadt (20.000 bis 100.000 Einwohner)
- o Kleinstadt (5.000 bis 20.000 Einwohner)
- o Landstadt/Landgemeinde (bis 5.000 Einwohner)

18. Was ist Ihr höchster Bildungsabschluss?

- o Kein Schulabschluss
- o Hauptschulabschluss
- o Realschulabschluss (mittlere Reife)
- o Fachhochschulreife (Fachabitur)
- o Allgemeine Hochschulreife (Abitur)
- o Berufsausbildung
- o Fachhochschulabschluss
- o Hochschulabschluss
- o Sonstiges:_________________________

19. Üben Sie aktuell einen Beruf aus?

o Nein, zur Zeit bin ich nicht berufstätig
o Nein, ich bin berentet
o Ja, ich arbeite Vollzeit als: _________________
o Ja, ich arbeite Teilzeit als: _________________

20. Haben Sie folgende Dokumente erstellt (mehrere Antworten sind möglich)?

- o Patientenverfügung
- o Vorsorgevollmacht
- o Anderes Dokument zur Festlegung meiner Wünsche für die Versorgung am Lebensende: ______________________________
- o Ich weiß nicht

21. Wissen Sie, was Palliativversorgung ist?

- o Ja
- o Nein
- o Ich weiß es nicht

22. Wissen Sie, was ein Hospiz ist?

- o Ja
- o Nein
- o Ich weiß es nicht

23. Haben Sie sich schon mal Gedanken über Ihre Wünsche für die Versorgung in der letzten Lebensphase gemacht?

- o Häufig
- o Gelegentlich
- o Selten
- o Noch nie

24. Haben Sie sich bereits mit Angehörigen über Ihre Wünsche für die letzte Lebensphase unterhalten?

- o Häufig
- o Gelegentlich
- o Selten
- o Noch nie

25. Wurden Sie bereits von jemandem auf Ihre Wünsche für die letzte Lebensphase angesprochen?

- o Häufig
- o Gelegentlich
- o Selten
- o Noch nie

26. Wenn ja, von wem wurden Sie auf Ihre Wünsche für die letzte Lebensphase angesprochen (mehrere Antworten sind möglich)?

- o Partner/in
- o Kind(er)
- o Freunde
- o Hausarzt
- o Urologe
- o Andere/r: ______________________________
- o Ich wurde bisher nicht angesprochen

27. Haben Sie mit Ihrem behandelnden Urologen über Versorgungsmöglichkeiten gesprochen, falls es zu einer Verschlechterung Ihres Gesundheitszustandes kommen sollte?

- o Ja
- o Nein
- o Ich weiß es nicht

28. Falls Sie dies nicht gemacht haben, hätten Sie Interesse an Gesprächen über die Versorgungs- und Behandlungsmöglichkeiten bei Verschlechterung des Gesundheitszustandes?

- o Ja
- o Nein
- o Ich weiß es nicht

29. An welchem Ort würden Sie „am liebsten“ versterben?

- o Zu Hause
- o Pflegeheim
- o Krankenhaus
- o Palliativstation
- o Hospiz
- o Im Haushalt von Angehörigen
- o Sonstiger Ort: ________________________
- o Ist mir nicht wichtig

30. Haben Sie Ihren gewünschten Sterbeort mindestens einem Angehörigen, ihrem Hausarzt oder Urologen mitgeteilt?

- o Ja, und zwar:
  - o Angehörigen/Freunden
  - o Hausarzt
  - o Urologen
- o Nein
- o Ich weiß es nicht

31. Sofern Sie eine Patientenverfügung haben, haben Sie Ihren gewünschten Sterbeort in Ihrer Patientenverfügung dokumentiert?

- o Ja
- o Nein
- o Ich weiß es nicht

Wir danken Ihnen sehr für die Zeit die Sie uns geschenkt haben und wünschen Ihnen für die Zukunft alles Gute!
